# Alginate-Based Composites for Corneal Regeneration: The Optimization of a Biomaterial to Overcome Its Limits

**DOI:** 10.3390/gels8070431

**Published:** 2022-07-10

**Authors:** Martine Tarsitano, Maria Chiara Cristiano, Massimo Fresta, Donatella Paolino, Concetta Rafaniello

**Affiliations:** 1 Department of Health Science, University “Magna Græcia” of Catanzaro Campus Universitario-Germaneto, Viale Europa, 88100 Catanzaro, Italy; martine.tarsitano@studenti.unicz.it; 2 Department of Experimental and Clinical Medicine, University “Magna Græcia” of Catanzaro Campus Universitario-Germaneto, Viale Europa, 88100 Catanzaro, Italy; mchiara.cristiano@unicz.it; 3 Campania Regional Centre for Pharmacovigilance and Pharmacoepidemiology, 80138 Naples, Italy; concetta.rafaniello@unicampania.it; 4 Section of Pharmacology “L. Donatelli”, Department of Experimental Medicine, University of Campania “Luigi Vanvitelli”, 80138 Naples, Italy

**Keywords:** alginate, tissue engineering, hydrogel, corneal regeneration, corneal endothelial disease, regenerative medicine

## Abstract

For many years, corneal transplantation has been the first-choice treatment for irreversible damage affecting the anterior part of the eye. However, the low number of cornea donors and cases of graft rejection highlighted the need to replace donor corneas with new biomaterials. Tissue engineering plays a fundamental role in achieving this goal through challenging research into a construct that must reflect all the properties of the cornea that are essential to ensure correct vision. In this review, the anatomy and physiology of the cornea are described to point out the main roles of the corneal layers to be compensated and all the requirements expected from the material to be manufactured. Then, a deep investigation of alginate as a suitable alternative to donor tissue was conducted. Thanks to its adaptability, transparency and low immunogenicity, alginate has emerged as a promising candidate for the realization of bioengineered materials for corneal regeneration. Chemical modifications and the blending of alginate with other functional compounds allow the control of its mechanical, degradation and cell-proliferation features, enabling it to go beyond its limits, improving its functionality in the field of corneal tissue engineering and regenerative medicine.

## 1. Introduction

The human cornea represents the outermost layer of the eyeball, and it shows relevant refractive and barrier functions. This membrane is avascular, and it looks like a clear hydrated gel whose transparency is mainly related to the presence of structural components that scatter incident light below the wavelength of the visible spectrum [1]. Along with the lens, the human cornea controls the entry of light, focusing the rays on the retina, thus contributing a major part of the eye’s focusing power [2]. This refractive process is similar to the mechanism from a camera for capturing an image, as the cornea with the lens of the eye can be thought of like a camera lens, while the retina is the film. In this way, if the image is not adequately focused, the film will be blurry; additionally, a loss of optical clarity will occur as a consequence of a damaged cornea [3].

**Figure 1:**
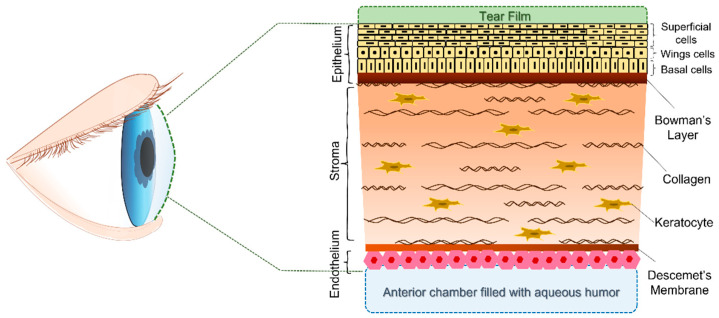
Schematic representation of the human cornea in cross-section. This avascular tissue is basically composed of three composite regions. The outermost anterior layer is the epithelium, which is followed by the stroma, which is the thickest layer of the cornea and is composed of keratocytes and collagen. The third portion is the endothelium, which is a monolayer of cells that lies adjacent to the aqueous humor. Bowman’s connective tissue layer and Descemet’s membrane are two acellular structures that connect the other portions of the cornea.

The corneal thickness is approximately 540 µm in the center and around 700 µm at the edges [4]. It is a heterogeneous tissue, structured in five parts: corneal epithelium, Bowman’s layer, corneal stroma, Descemet’s membrane and corneal endothelium (Figure 1) [5]. Each layer has specific roles, contributing to the proper functioning of the cornea. The epithelium, which is the outermost layer of the cornea, can be further separated into three layers of cells: superficial cells, wing cells and basal cells [4]. Superficial cells limit the passage of microorganisms and toxins, due to the presence of tight junctions between them, whilst basal cells are engaged in epithelium renewal. Indeed, corneal epithelial stem cells, located at the limbus (or corneoscleral junction), move in a centripetal direction, multiplying asymmetrically and completing the epithelium renewal process within one week [6,7,8]. Bowman’s layer is interposed between the basal cells of the epithelium and the near corneal stroma; if damage occurs to this layer, it does not regenerate, but no relevant structural change occurs in the cornea, so it is supposed it could participate in the protection of deeper structures [9]. Contrariwise, the stroma, which is composed of collagen fibrils, has a key role in the corneal structure, because it is responsible for the strength of the cornea and the maintenance of the shape, and it represents ~90% of the corneal architecture [10]. The presence of numerous proteoglycans associated with glycosaminoglycans and sulfates ensures the clearance and continuous hydration of this corneal layer. At the same time stromal cells, namely keratocytes, contribute to the renewal of this layer, thus defining the stability of the stromal scaffolding and assisting any wound healing processes [11]. Descemet’s membrane is tightly attached to the posterior corneal stroma, and it is implicated in the maintenance of the below endothelium structure [10]. Microscopic analysis of this layer has shown that it is characterized by a discontinuous and porous nature. Fibrils of collagen, mainly types IV and VIII, as well as fibronectin and laminins, compose the architecture of this interfacial matrix, and they are organized into a hexagonal lattice structure covering the corneal endothelium, a single layer of hexagonal cells [12,13]. The gaps and tight structural junctions, which are typical of this monolayer, allow the passage of water between the aqueous humor and the stroma to control the hydration state of the stroma and prevent the formation of edema, which would lead to a reduction in vision. This role is defined by the presence of Na^+^/K^+^ ATPase pumps, which is located in the basolateral portion of this membrane, allowing ions and water to move to the hypertonic aqueous humor from the stroma, contributing at the same time to the transfer of nourishment sources to the cornea [14,15].

### Corneal Diseases: Current Strategies and Their Limitations

Considering its anatomical position, the human cornea is often exposed to chemical and physical stresses that can lead to its structural alterations [16]. In accordance with the last global report on vision, presented by the World Health Organization, over 2.2 billion people live with a vision impairment; among them, 123.7 million people are affected by unaddressed refractive errors and 4.2 million are suffering from corneal opacities [17]. Keratitis [18], corneal dystrophies [19], wounds [20], neovascularization [21] or structural modifications in thickness [22] are just some examples among the best-known diseases affecting the cornea. When irreversible damage involves the cornea, a loss of corneal transparency occurs, leading to severe vision impairment or blindness.

For several decades, the only option for treating corneal blindness has been the full penetrating keratoplasty (PK) from cadaveric donors [23,24]. However, PK can cause further complications as transplant rejection, astigmatism, uveitis, retinal detachment or corneal ulceration depending on the reopening of surgical wounds [25].

Nowadays, developments in surgery technologies allow transplanting only some corneal layers in the case of patients who have partially damaged cornea, permitting them to retain their healthy and functioning portion of membrane and to reduce the amount of allogenic tissue used [26]. This is the case of patients suffering from Fuch’s dystrophy, which results in a gradual thickening of Descemet’s membrane and reductions in cell density. Once cell density reaches values of 500 cells/mm^2^, the endothelium seems to be no longer able to pump sufficient fluids out of the stroma, causing swelling and visual damage. In this context, if the patient has no other compromised corneal structures, the disease can be solved through a selective Descemet’s membrane endothelial keratoplasty (DMEK) [27,28]. This strategy is clearly beneficial for patients as it allows the minimization of the phenomena of rejection and cases of therapy failures, compared to treatment with penetrating keratoplasty, which is, however, still necessary in case of more extensive damage [29].

Despite the great efficacy of transplantation strategies, it must be pointed out that very few donors are recorded per year (around 130,000 corneal donors) and most of them are not deemed eligible for transplantation, so many patients awaiting transplant often remain untreated [30]. This relevant gap between donor tissue demanders and suppliers also results from corrective eye surgery, which makes patients’ cornea unsuitable for allografting; thus, different strategies have been proposed, including limbal autografting, which consists of a transplantation of a graft harvested from the limbus of the patient’s healthy eye or simple limbal epithelial transplantation [31] from the patient’s healthy eye to be transplanted onto the damaged cornea [32].

In some cases, even penetrating keratoplasty is not enough to restore the functionality of the cornea, such as in the case of patients having limbal stem cell deficiency (LSCD). In those cases, no growth will occur on the engrafted tissue from the patient’s own epithelium, and the treatment will probably fail; then the injection of cultured limbal stem cells into the cornea could be required before performing any other surgical procedures [25]. However, in this case, some critical issues limit the use of stem cell transplantation as an eligible therapeutic strategy, like the identification of selective markers to isolate specific stem cell populations [33] or differences among donors of mesenchymal stem cells (MSCs) in terms of age, weight, genetic or clinical history that can compromise treatment [34,35]. Another major drawback in the use of cell therapy arises in the different methods used for the isolation and culture of cell lineages, which leads to different results [36]. Finally, several other studies demonstrated that MSCs lack “immune privilege”, so they may not be sufficiently safe, although this aspect is considered to be negligible in the case of the corneal tissue that is self-immune privileged [37,38,39]. In light of these limitations, the research is still ongoing to perfect cell therapy.

As for the limits of the transplantation of cornea, the scientific community is focusing attention on suitable alternatives, pointing out the need to replace the biological membrane with functional materials or to induce the regeneration of the native cornea in patients [26,40,41,42]. Tissue engineering represents a promising approach to address this goal. In 2014, a great result was achieved as the European Medicines Agency (EMA) authorized the use of Holoclar in the European Union [43]. This treatment, first proposed by the Italian company Holostem Terapie Avanzate S.r.l. (Modena, Italy), is characterized by ex vivo expanded autologous human corneal epithelial cells containing stem cells. Holoclar represents the first innovative therapy containing stem cells approved in Europe, and it was thought to be useful for patients with moderate to severe limbal stem cell deficiency, unilateral or bilateral, caused by chemical or physical ocular burns. The patient’s limbal cells are harvested from the corneal edge (at least 1–2 mm^2^ of undamaged limbus are required for biopsy), amplified and transplanted onto the damaged area. The great results obtained from the monitoring of this “tissue engineered product” highlighted the need for further advancement in regenerative medicine in the field of corneal diseases [44].

The necessary conditions to be adopted for the use of this innovative engineered strategy emphasize the need for artificial alternatives, to which “keratoprosthesis” refers [45]. Looking more closely at the complex framework of the cornea (see Section 1), it appears that it is impossible to recapitulate using the current available tissue engineering strategies and materials. However, it may not be necessary to exactly match the biological structure as long as the scaffold respects the properties of transparency, is able to accommodate cells and is characterized by sufficient bioadhesive properties.

In light of these considerations, this review discusses the use of alginate, a naturally derived material, as a valid alternative to be used in tissue engineering for corneal regeneration. The chemical modifications and the linking of alginate to other functional compounds were also reported to investigate their role in exceeding the limits of pure alginate.

## 2. Material Properties to Match the Corneal Microenvironment

Since the first keratoprosthesis surgery was performed by A. von Nussbaum [46], several synthetic materials (e.g., poly(methylmethacrylate), poly(urethane), poly(tetrafluoroethylene)) have been employed as artificial implantable devices but with limited success in clinical outcomes, due to postoperative complications (rejections from host tissue, obstacles to vision and persistent epithelial defects) [47,48,49]. Hydrogels derived from reticulated polymers with a high grade of water-swelling ability represent favorable and innovative alternatives to plastic-based implants, thanks to the homogeneous distribution of their cargo derived from their non-invasive interreducibility in the eye [50].

On the other hand, great interest has arisen in naturally derived biomaterials as potential constituents of scaffolds in corneal field of tissue engineering and regenerative medicine (TERM).

Hydrogels are polymeric networks characterized by the peculiar ability to swell in water, thanks to the hydrophilicity of the constituent polymeric chains, but without completely dissolving in aqueous medium. This feature allows them to be considered an “ideal class of materials for biomedical application”, as hydrogels can incorporate high water content, mimic te natural living soft tissue and entrap in their grid bioactive molecules and cells [51]. The physico-chemical properties of hydrogels can be tunable depending on the fabrication method and are strongly related to the crosslinking methods chosen [52].

Mostly, whatever the field of application of a biomedical hydrogel and whatever the origin of its components (synthetic or natural), the choice of material is the key-factor in the realization process of a product. The physico-chemical, mechanical and technological features of a biomaterial determine how friendly with the tissue the construct is, ensuring or not the repairing or regenerative purposes.

Mechanical and viscoelastic behavior, biodegradability, low immunogenicity, bioadhesiveness and transparency represent the essential biocompatibility characteristics to be considered in the use and optimization of a biomaterial, and none of these features can be overlooked or missing in the design and development of a biomaterial for ocular regeneration [53]. All of these parameters should also be considered and eventually modified in light of the fabrication techniques utilized for the realization of the scaffold, such as 3D-bioprinting or electrospinning [54,55,56].

Biocompatibility is commonly recognized as the characteristic of a material fulfilling its biomedical purpose (both therapeutic, reparative and regenerative) without causing unwelcome local or systemic responses in the host and to co-exist in contact with the human body without disturbing the physiological tissue balance of the administration site [57,58,59,60]. The re-evaluation of this view was well explained by Williams, who reminded us that biocompatibility has to be defined primarily as a function of the application site and the situation in which the material is used [61]. Indeed, to be suitable in an ocular environment, the ideal requirements for a material for corneal regeneration must include: (i) the ability to stimulate or assist extracellular matrix (ECM) synthesis; (ii) specific mechanical stability properties; (iii) optical transparency and proper refractive index; (iv) good features for corneal cell adhesion, support and survival when present as incorporated elements into a bio-scaffold; (v) a manageable degradability profile and the limited induction of an immune response by the material itself or by its derived degradation products; (vi) the ability for oxygen and nutrient transfer through its 3D-structure [62]; and (vii) high adhesion to the native tissue [63].

An ideal material should be as similar as possible to the ECM matrix to maintain tissue homeostasis and to encourage tissue development. Several synthetic polymers (e.g., poly-ε-caprolactone (PCL) [64], poly-L-lactic acid (PLLA) [65], polyvinyl-alcohol (PVA)) have been used in the TERM field, thanks to their derived ECM-like structures and the possibility of manufacturing scaffolds with high-controlled porosity. Unfortunately, while PVA alone is able to guarantee transparent film and hydrogel-scaffolds, some of the aforementioned polymers (PCL, PLLA) are considered not suitable for a corneal address, due to the lack of transparency [66]. Typically, this class of polymers do not represent a great immunological hazard, specifically because they do not possess biologically functional domains. Although it might seem to be an advantage, if considered from another perspective, the absence of reactivity with biological molecules (such as growth factors) will strongly limit cell adhesiveness, as well as the possibility of cells’ survival both within the raw material and at the site of application [67]. Even if there are conflicting theories about natural polymers and their immunogenicity potential, many of them (polysaccharides, proteins and polyesters) have their own similarity to ECM thanks to their synthesis by living organisms [68]. This feature gives them a great advantage in cyto-compatibility and support but without losing the possibility of obtaining porous and bio-adhesive matrices [69].

In recent years, great attention has been given to acellular corneal ECM, derived from different decellularization techniques, thanks to its ability to maintain the composition and structure of the native cornea. Del Barrio et al. [70] demonstrated how coating with ECM-proteins (collagen-keratan sulfate) of synthetic ethyl acrylate (EA) copolymers provides for the better biointegration of the scaffold with the surrounding corneal environment, optimizing human adipose-derived adult stem cells (h-ADASC) adhesion compared with the naked material. In this experimental work, the researchers discarded methacrylic-acid-based copolymers (MAAc) for the in vivo assay because of their biophysical instability but mainly due to their lack of flexibility and transparency.

Transparency, like other features of corneas such as strength and morphology, is attributable to the anatomical structure of the corneal stroma, which represents 90% of the corneal thickness. The loss of transparency and the consequent reduction or loss of vision are the most common consequences of corneal disease [71,72]. It is easy to deduce that any biomedical implant, focused on repairing damage to the eye, must be transparent and do not change the refraction light capacity. Transparency in corneal regeneration is usually assessed by the determination of optical transmission, so hydrogels with optical transmission ≥90% can be considered transparent, ≤90% but ≥10% as translucent and ≤10% as opaque [73]. Moreover, the finished product must maintain the optical properties until a complete degradation of the biomaterial occurs, or it will be removed from the eye (in the case of ocular inserts).

In their in vivo investigation of the effects of acellular porcine corneal stroma sheets (APCS) with or without keratocytes after transplantation in a rabbit lamellar keratoplasty (LKP) model, Ma and coworkers [74] scored the animals’ corneal opacity at 6 months post-operation. They found that serious corneal opacity occurred when those sheets were implanted without incorporating cells, and this result allowed them to validate the system seeded with keratocytes, which did not create obstacles to transparency and transmission of light until 6 months after the implantation. Other efforts to form a multi-layer 3D architecture system based on overlapping silk films seeded with corneal stromal stem cells, such as that of Ghezzi et al. [75], were instead unsuccessful, because a reduction in the transparency of the scaffold was observed when compared to single films.

Even if transparency and biocompatibility features are often achieved, the mechanical unsuitability of a biomaterial may prevent its forward travel to clinical translation. The main actor in attributing mechanical properties to the external eye section, such as strength, viscosity or elasticity, is once again the corneal stroma [76]. The architecture of the stroma exactly reflects each of these assets. Collagen confers strength and elasticity, while cellular components and proteoglycans are responsible for viscoelastic features [77]. So, during the characterization of a corneal construct or 3D-scaffold, the most commonly considered mechanical properties are the Young’s modulus and the ultimate tensile strength. Young’s modulus is a measure of the stiffness/resistance or elasticity of a material, as a consequence of its opposition to or reversibility in deformation, when it is subjected to any load [78]. On the other hand, tensile strength is the force that must be applied to a material to cause it to break [79]. The human cornea does not have a unique value of Young’s modulus, because it changes with the characterized region (the anterior region is stiffer than the posterior one) or with the type of measurement assessed. Therefore, this value could oscillate between 0.1 to 57 MPa, while the tensile strength of the cornea is commonly considered to be around 3–6 MPa [75,80,81,82]. For sure, damage to corneal superficial or deeper layers will lead not only to a poor biomechanical support and protection but will also be responsible for the altered permeability of nutrients into the central cornea, thus hindering local cell homeostasis and viability [83].

Since Madden et al. [84] proposed silk fibroin membranes to culture corneal endothelial cells, the protein was widely investigated in the field of corneal transplants, thanks to suitable transparency and mechanical properties [85]. However, to enhance he cell adhesion and proliferation, in several works, fibroin had to be blended with other materials [86,87,88,89]. In particular, ocular films made by a particular silk fibroin, derived from *Antheraea mylitta*, which contains a natural aminoacidic sequence (Arg-Gly-Asp), demonstrated a high permeability to metabolites and nutrients [90]. Often this feature is not easily addressed, thus requiring the addition of other substances that can improve both nutrient/oxygen permeability and cell attachment and growth (collagen, gelatin, tropoelastin) [91,92]. The use of naturally derived biomaterials, like those mentioned above and also the amniotic membrane (AM), always carries with it the risk of an unwanted immune response that could cause rejection as well as implant failure. In their experimental work, Qi et al. [93] proposed an engineered AM as a vehicle for the in vitro cultivation and transplantation of limbal epithelial stem cells. Once the construct was obtained, they were surgically transplanted onto 15 patients (16 eyes), who experienced thermal or chemical eye burns. After a complete ocular examination was performed, the study recorded 12 positive outcomes, due to a complete reconstruction of the ocular surface achieved in 12 months. The remaining four eyes underwent immune rejections due to elicited immune response that completely degraded the AM, so the patients re-experienced recurrent corneal opacity and neovascularization [93].

Hydrogel bio-adhesiveness is ruled by its hydrogen bonding ability and then strongly influenced by the structural arrangement of the raw materials that compose it. Some hydrogels based on natural polymers, such as chitosan and chitin, showed notable adhesion and an appreciated biodegradation profile, thanks to the presence of hydrophilic functional groups, which allow the formation of hydrogen bonds [94,95]. These features of chitosan/chitin could be enhanced or managed with some modification. For example, Shou et al. [96] proved the superior tissue-adhesion potential of a catechol-hydroxybutyl chitosan (HBCS-C) thermoresponsive hydrogel, as a new insight into hemostatic agents, instead of conventional chitosan-based ones. Isobe and co-authors [97] preferred chitin to chitosan as a material for ocular purposes, because the absence of positively charged amino groups makes its derived hydrogel easily degradable in vivo by lysozyme. The combination with other biomaterials such as PVA and *n*-hydroxyapatite (*n*-HA) made chitosan an interesting candidate for cornea tissue engineering applications. Liang et al. proposed this blending to obtain a composite hydrogel as an artificial corneal scaffold, by joint chemical/physical crosslinking [98]. However, the achievement of reliable results with chitosan for ocular delivery and regeneration is often linked to the use of chemical crosslinking agents such as glutaraldehyde, which is not free from potential toxicity, irritation and sensitization [99,100,101].

In the panorama of biomaterials investigated for the tissue regeneration of the cornea, each of them has one or more individual advantages, but, unfortunately, these are often not enough to fill all the needed features for the desired product. Therefore, continuous strategies of the modification, functionalization or combination of materials are necessary to converge on better production and efficacy outcomes.

## 3. Advantages and Limitations of Using Alginate for Corneal TERM

Among the wide range of natural biomaterials employed in tissue engineering, a relevant position is taken by alginates [102]. This class of naturally derived substances are largely considered biocompatible, non-toxic, non-immunogenic and biodegradable [103], but all of these qualities are related to the materials’ own specific features of molecular structure, type of living organism they derived from and their fabrication process. In recent years, alginate has been largely employed in the pharmaceutical field for the known advantages derived from its application, both as excipient for drug delivery systems and in regenerative medicine as the main material for hydrogels and scaffolds [104,105,106,107,108,109,110,111].

### Structure and Factors That Influence the Gelation of Alginates

Alginate is a natural heteropolysaccharide abundantly present in numerous species of brown algae (e.g., *Ascophyllum nodosum*, *Macrocystis pyrifera* and *Laminaria hyperborea*), as well as it is synthesized by some bacteria (e.g., *Pseudomonas aeruginosa* and *Azobacter vinelandii*). Alginates are capable of such protection and mechanical-resistance features in several contexts [112,113] For example, alginate supports and strengthens the structure of brown algae, as it is present in cell walls, giving algae flexibility and resilience, necessary for plant growth in the sea and to counteract strong ocean currents. In bacteria, alginates are secreted to constitute part of the protective capsule as a fundamental constituent, thanks to their role in bacterial adherence, colonization and survival in the infected host organism [114,115].

**Figure 2:**
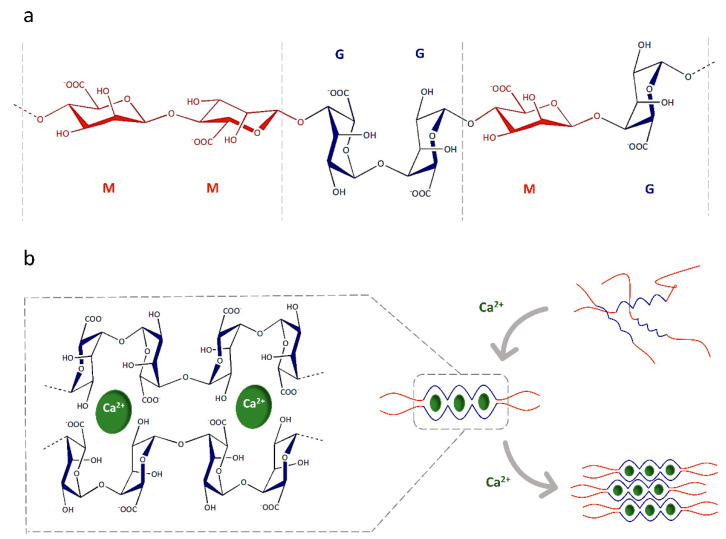
(**a**) Schematized representation of chemical structure of alginate, composed of 1,4 α-l-guluronic acid (G) and 1,4 β-d-mannuronic acid (M) residues, arranged in homo- (MM, GG) or -hetero (MG) blocks. (**b**) The junction zone of the “egg-box” model during ionic gelation in the presence of divalent cations (e.g., Ca^2+^).

The aforementioned functions are just the reflection of the intrinsic characteristics of this extracellular polysaccharide, which differs from its class analogues by having a relatively simple structure. The molecular structure of this polysaccharide includes units of 1,4 α-l-guluronic acid and 1,4 β-d-mannuronic acid, respectively called G and M residues (Figure 2a). The ratio of M/G residues and the composition in terms of homo-(MM, GG) and hetero-(MG) polymeric blocks depend on the natural sources from which the alginate is extracted. Differences in arrangement are strongly responsible for the variations in the main physico-chemical characteristics of alginates [116], including gelation and hardness [117,118,119], when in contact with crosslinking agents but also the resultant immunogenicity [120,121]. It was assessed that alginate with a high percentage of GG blocks forms stiffer and more inflexible hydrogels, precisely because the carboxyl groups of residue G are responsible for the crosslinking with divalent cations such as Ca^2+^, Zn^2+^ and/or Mg^2+^ [122,123], forming the well-known “egg-box” model [124,125] (Figure 2b).

Quite the opposite, when M units abound, the resulting products are softer and more elastic, but it has been extensively demonstrated that they have greater immunogenic potential [126,127]. The molecular weight of the commercially available form of alginate (sodium alginate, Na-Alg) can fluctuate between 32 and 400 kDa, depending on the source, species and extraction process [128,129]. In addition to the molecular structure, the molecular weight also significantly impacts alginate viscosity and its gel-formation properties, such as swelling and shrinking ability, along with its biological activity [130]. The molecular weight, the M/G residues molar ratio and the viscosity of the derived hydrogel also fluctuates according to the method used to purify polysaccharides like alginates [131,132,133]. This purification step is necessary for any biomedical application for alginate gels. Depending on their derivation and the purpose for which their use is intended, alginates can be purified by various techniques. Among these, the most commonly used are purification by precipitation, filtration or extraction, but exclusion chromatography and chemical purification must also be mentioned. The aforementioned purification methods allow the removal of protein contaminants, endotoxins and polyphenols, normally present both in commercial and extracted-from-brown-seaweeds alginates, which could lead to an exacerbated immune response in the host, reducing the biocompatibility of alginate-based scaffolds [120,134]. In 2019, Torres and coworkers characterized alginate from a commercial source and isolated sodium alginate from the blade and midrib of *Undaria pinnatifida*, before and after purification. They investigated the toxicity and biocompatibility of the extracted alginate, both on macrophage-like cell lines and on bone marrow stromal cells. From the analysis of cell morphology, viability and differentiation results suggested that the impurities present in the extracts of alginates induced toxic effects that could be completely avoided by a simple purification step [135].

Compared with lower-molecular-weight, high-molecular-weight alginates exhibit easier and faster gelling and enhanced elasticity. Managing molecular weight is key to independently controlling the alginate pre-gelling solution viscosity, as well as the post-gelling stiffness. For this reason, if a suitable combination of high and low molecular weight is chosen and the gelation rate is controlled, the mechanical properties of ionically crosslinked networks can be modulated [136], in addition to the cell encapsulation ability [137,138], thus making alginates suitable and attractive materials for corneal tissue engineering.

Although covalent cross-linking hydrogels would allow the better stability of the systems, ionic gelation still represents the preferred way to form alginate hydrogels intended for TERM, due to its easily reversible process that does not involve chemical covalent crosslinkers, which may be toxic [139]. Therefore, alginate hydrogels have been investigated in different biomedical applications, for example in cardiac and in cartilage TERM, mainly because of their remarkably similar features to the ECM of human tissue [140,141].

## 4. Alginate Composite

**Figure 3:**
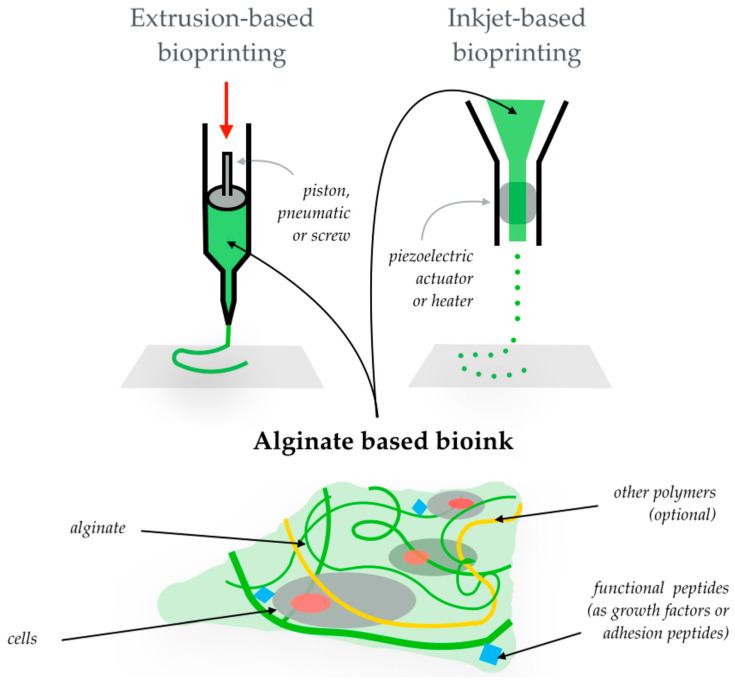
Bio-printing techniques used to obtain an alginate-based 3D-hydrogel. Re-adapted from Axpe, E.; Oyen, M.L. Applications of alginate-based bioinks in 3D bioprinting. *Int. J. Mol. Sci.* **2016**, *17*, 1976. [145].

As reported in Section 2, in corneal TERM, the chosen material must have specific features to fulfill the regeneration purpose. Unmodified alginate alone has some of them, such as biocompatibility, the absence of immunogenic potential, transparency, high swollen state, and bioadherence with host tissue, but, at the same time, it lacks suitable post-swelling mechanical features, opportune cell adhesion properties and a controllable biodegradation rate [102,139,142,143] For those reasons, the main functionalization and blending techniques, carried out with the aim to fill or improve the characteristics of alginates within the corneal TERM, are summarized in Table 1 and discussed in the subsequent sections. The offered strategies included techniques such as electrospinning [55] and bioprinting (Figure 3), as they stand for innovative manufacturing methods to create hydrogel-based scaffolds alongside the more conventional injectable or in situ formation methods [144].

### 4.1. Combinations to Reinforce Alginate Hydrogel-Based-Scaffolds

Even if different hydrogels based on natural polymers are attractive in corneal scaffold manufacture, thanks to their ability to incorporate high content in water and their similarity with ECM, many of them still miss a highly organized fibrous structure [151,156], which can provide the right three-dimensional environment for cell infiltration and/or delivery.

As previously reported by Wu et al. in 2012, the more faithful the reproduction of the precise spatial organization of the engineered corneal tissues, the more similar the outcomes of corneal strength and optical properties [157].

Although alginate hydrogels are characterized by optimal transparency and tunable mechanical properties depending on M/G ratio and from the degree of cross-linking [130,158,159,160], they lack a precise spatial organization.

Strange and co-workers explained the potential of combining different materials to manage their toughness and to create a composite that is able to better fit the native tissue [146]. They investigated the wild range of mechanical properties of polycaprolactone (PLC) as electrospun fibers when combined with alginate hydrogels (1%, 3% and 5%). When backfilled with alginate, the resultant samples underwent an increase in thickness from 1.91 ± 0.76 to 2.96 ± 0.45 mm (for PCL-1%Alg), up to 5.43 ± 1.06 mm when 3% alginate was used in hydrogel. The highest concentration of polysaccharide led to hydrogels with a non-uniform shape that were discarded. After mechanical properties testing, fiber-reinforced hydrogels appeared more robust to failure than pure alginate hydrogels. Even the PCL-3%Alg sample, which showed a tensile modulus comparable to the 3% pure hydrogel, was characterized by a strength ten times greater than 3%Alg. Alginate composites also remarkably enhanced their strain-to-failure property with respect to pure material, so this effect was marked due to the presence of PCL [146].

These findings could be translated to the corneal TERM field, taking into consideration the efforts made by Tonsomboon and Oyen in forming a new alginate composite with gelatin [148]. The authors chose as the blending agent porcine skin gelatin, a protein resulting from the hydrolysis of collagen, exploiting its low cost and electrospinnability in water-based co-solvents [161]. They conducted mechanical characterization by uniaxial tensile testing, and, since often the reduced transparency is associated with a non-uniform orientation of fibers [162,163], they also performed an optical test on the light transmitted from the hydrogels, comparing the values with those of the porcine cornea. The resulting hydrogels’ thickness increased as a function of the increasing thickness of the gelatin mats used. Moreover, before the combination with gelatin fibers, the 3% alginate hydrogel was slightly nonlinearly elastic, while, after the addition of electrospun gelatin, its tensile properties were substantially improved. The tensile elastic modulus and tensile strength values before reinforcement with aligned gelatin fibers were 77.88 ± 18.67 kPa and 19.29 ± 9.00 kPa, respectively. After the combination, they turned into 0.50 ± 0.11 MPa and 0.34 ± 0.03 MPa, values which were almost doubled when the cross-linking process was assessed in an ethanolic solution. Even though these results emphasized the exciting potential of the composite, thanks to its enhanced mechanical properties, the optical properties of the hydrogels did not completely match the optical transparency required by corneal native tissue [148].

A few years later, Stafiej and colleagues included nanofibers of PCL into alginate networks for corneal wound healing application, characterizing both random- and aligned-fibers-loaded hydrogels [147]. They obtained thickness values of swollen constructs (80–110 µm) remarkably similar to that of amniotic membranes (20–100 µm). The research group achieved an enhanced suture retention strength for random-nanofibers-enriched hydrogels, which was comparable with the gold standard amniotic membrane and directly proportional to increasing nanofiber web thickness. Unlike in the previously described works, here the authors achieved the set goal, without losing the transparency of the material, even if results were inversely proportional to the thickness of the scaffold. Indeed, the less thick scaffold (5–10 µm) showed results comparable to the pure alginate hydrogel, allowing high readability with the “readable font size method” [147].

Transparent equivalents of corneal stroma were generated by Isaacson et al., using the pneumatic extrusion technique of bioprinting, thus combining sodium alginate with methacrylated collagen in different ratios [148]. The corneal substitutes were prepared starting from low-viscosity bioinks (two composed only of 3% alginate + 8 mg/mL methacrylated collagen and four made of 2% alginate + 6 to 8 mg/mL methacrylated collagen, named from Coll-1 to Coll-4). Among the six bioinks formulated, the one composed by one part 8 mg/mL to two parts alginate showed the most suitable printability and stability after bioprinting, as well as the best preservation of corneal shape.

### 4.2. Strategies to Improve Cell Incorporation and Survival into Alginate Hydrogels

Alginate, as an anionic compound with several carboxylic end-groups, is able to swell when it is hydrated and to interpenetrate its chains with the chains of mucins, which constitute a coating on the ocular surface. This mechanism allows the ocular adhesiveness of alginate that prologue the precorneal residence time of the pharmaceutical formulations based on it [164]. Due to the alginate’s deficiency of cell adhesion motifs, its muco-adhesiveness is not assisted by great cell-adhesiveness, thus representing a limitation in hydrogels’ biomedical application [165].

**Figure 4:**
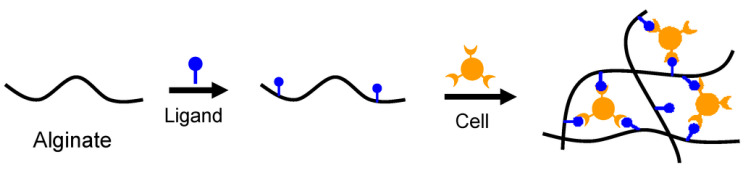
Representative mechanism of cells’ inclusion in an alginate network after coupling with a ligand, such as RGD cell-adhesion motif. Re-used from Sun, J.; Tan, H. Alginate-Based Biomaterials for Regenerative Medicine Applications. *Materials* **2013**, *6*, 1285–1309. [102].

One way to improve this feature is to functionalize alginate with native ECM-derived materials, such as collagen and its resultant gelatin [166]. Gelatin is a biodegradable protein, produced by a denaturation process of collagen, that leads to the exposure of the RGD (Arg-Gly-Asp) cell-adhesion motif [167,168] and that acts as a cell attractant, as shown in Figure 4, promoting epithelialization and granulation tissue formation [169,170,171]. For several years, the coupling of integrin-binding peptide sequences, such RGD, has been demonstrated to be useful in improving cell attachment to calcium alginate hydrogels by Rowley and Mooney [172,173].

In 2016, Yan et al. proposed an easy and low-cost process to generate a contiguous viable cell sheet, using human corneal epithelial cells, by the functionalization of sodium alginate with RGD, prior to forming a calcium hydrogel [150]. In details, they incubated the RGD-alginate hydrogel in a culture medium until a suitable number of cells attached to the 3D network and then recovered a cell sheet just relying on the gel-sol transition of the calcium alginate hydrogel in the presence of sodium citrate as a chelating agent. They investigated both 5% and 13% degree of RGD substitution and, by means of brightfield images of cells taken 24 h following seeding, the extent of cell attachment to RGD hydrogels was observed, with a noticeable increase in the 13% degree of substitution. Sheets with high viable human corneal epithelial cells were retrieved anyway [150].

This technique appears particularly useful to avoid the uncontrolled production of ECM by seeded cells in hydrogels, which could fill the pores and limit the migration of incorporated cells [174].

Isaacson et al. also achieved reliable results in terms of cell incorporation and viability through their previously discussed 3D-printed cornea-like structure made of alginate and methacrylated collagen (see Section 4.1). In detail, they observed great cell viability on day 1 post-printing (almost 92%), that remained high after 7 days (around 83%), without the formation of cell aggregates [149].

Recently, an interesting work by Farasatkia and Kharaziha suggested micropatterned membranes for corneal stroma tissue engineering [151]. This construct was born with the aim of merging in a double layered system the transparency and bio-adhesiveness abilities of alginate with the already proven combination of silk nanofibril/methacrylated gelatin, characterized by good wettability and adjustable mechanical properties [92]. Initially, they produced these nanohybrid hydrogel solutions based on silk and methacrylated gelatin and used a silicone mold (with two different groove sizes) to imprint onto the hydrogels a micro-pattern, which mimics the structure of native cornea. After UV crosslinking and drying, a transparent film was obtained. Contemporaneously, an alginate solution was prepared, loading ascorbic acid (in 0.2, 0.4 and 0.6% of concentration) prior to ionic crosslink and drying it to obtain a second film. The two films were immersed in water until completely swollen and linked together to obtain a double layer construct, which was seeded with human corneal stromal cells.

An adhesion test of the construct in humid conditions measured on sheepskin showed comparable adhesion strength between double layered films and commercial corneal glues, based on cyanoacrylates and fibrin [175]. The elastic modulus of combined layers was almost four times greater than those of single films, and the transmittance showed more suitable values for constructs obtained with narrower-width mold grooves. The cell viability on films was figured out by an MTT test, showing no cytotoxicity and an increase in survival until 7 days. The cell survival was higher (68.5 ± 7.5%) in double-layer film with ascorbic acid than the empty one (51 ± 12%). Thus, the authors concluded that ascorbic acid sustained the proliferation of stroma cells, as previously reported by Shah et al. [176]. Furthermore, regarding cell viability, the use of smaller pattern size (about 50 µm) led to the highest cell survival, as confirmed by fluorescent microscope images that revealed above 96% cell viability for all samples, thus highlighting a strong interaction between silk/gelatin/Alg film and cells. More than 90% of cells in the film obtained by the smaller pattern mold were located at angles close to the vertical axis (between 0 and 20 degrees). Indeed, the work by Farasatkia and colleagues can be considered an important advance in the corneal stroma regeneration field, especially for the achievement of cell orientation with micro-patterning techniques [151].

In 2019, Xu et al. underlined the absence of an optimal scaffold to transplant limbal stem cells (LSCs) to promote corneal restoration after corneal alkali burns [152].

Effectively, even if several research groups, such as Huang et al. [177] and Tsai et al. [178], worked with hydrogels to improve drug delivery strategies in the treatment of corneal wound healing, few studies have attempted to deliver LSCs. Then Xu et al. proposed an in situ hydrogel of alginate and chitosan, to investigate its potential in LSCs delivery. After the absence of cytotoxicity of the hydrogel was assessed on rabbit LSCs in vitro by and its degradation profile was investigated in vivo on mice, they performed in vivo studies on a rabbit alkali burn model. The novel hydrogel was able to completely reduce corneal opacity within 28 days after an induced alkali burn, but even after 7 days, the reduction of opacity was markedly visible compared to the untreated model group. The immunofluorescence analysis of epithelial and stromal cells, marked with K3 + 12 and vimentin respectively, suggested that the combination of LSCs and composite hydrogel strongly promotes corneal injury repair [152].

### 4.3. Oxidized Alginate to Control Alginate Degradation Rate and More

Ionically cross-linked alginate hydrogels degrade slowly and in a non-linear manner through the exchange that occurs between calcium ions (or other divalent ions) and physiological ions such as Na^+^ [179]. This process is responsible for the unpredictable release of both low-molecular-weight and high-molecular-weight strands of alginates. Only the strains with a molecular weight below 50 kDa are easily removed from the body through the kidneys. Even if biodegradability rate can be tuned by changing the composition and molecular weight of the polymer, the lack of alginate-degrading enzymes in mammals represents the main obstacle, because no hydrolytic or enzymatic chain breakages of higher-molecular-weight and longer chains of alginate occur under physiological conditions in mammals [180]. One possibility to accelerate the biodegradation of alginate is exposure to gamma irradiation. In particular, low radiation doses (<8 Mrad) can be exploited to induce the formation of glycosidic bonds between MM and GG blocks, which will shorten alginate chains but without a significant alteration in block content [181].

Another approach, experimented on by Wu et al., consists of the use of sodium citrate, whose citrate ions chelate calcium ions of alginate ionically crosslinked hydrogel, creating calcium-citrate complexes [153]. This process was proven to be effective in dissolving calcium chloride crosslinked alginate hydrogels and was already used by the aforementioned Yan and colleagues for other purposes [144,150]. Wu et al. applied this strategy on 3D-printed constructs made by alginate, gelatin and collagen, laden with human corneal epithelial cells to manage with a degradation-controllable scaffold. From an analysis of sodium citrate effects on hydrogel degradation, it has been found that, if the citrate/alginate molar ratio is higher than 1, the hydrogel will completely degrade in less than 3 days. By reducing the mole ratio, they were able to obtain degradation prolonged until 2 weeks. The optical density was not affected by the presence of sodium citrate, which on the contrary elicited cell proliferation from day 2 to day 8 of the study. Summarily, they demonstrated that the degradation effect of sodium citrate provides a faster growth of human corneal epithelial cells in the 3D hydrogel, having an increasing ability in proliferation [153].

A further modification that deserves to be mentioned is the oxidation of alginate, a method employed to trigger the hydrolysis process. One of the main oxidizing agents used for alginate is sodium periodate, which preferably reacts with G units but can also oxidize M ones. The effect of oxidation on degradability, as well on swelling behavior and viscoelastic properties, can be controlled, depending on the degree of oxidation.

The investigations about biomedical applications of oxidized alginate (OA) are continuously incrementing and generating curiosity [42]. For example, the partial oxidation of alginate allowed the provision of the controlled degradation kinetics of hydrogels and also to manage the release of incorporated factors, as is also demonstrated in other tissue regeneration fields [182,183].

In corneal healing and regeneration, the oxidation of alginate represents a promising modification of the material’s properties that makes it more suitable to meet the requirements for the ocular administration site. Xu and coworkers used OA in the aforementioned work, thus experiencing no issues regarding the degradation of the hydrogel combined with carboxymethyl chitosan [152]. Liang and colleagues proposed an in situ biodegradable hydrogel by the self-cross-linking of sodium-OA and chitosan, evaluating cyto- and histo-compatibility [154]. Assessing the absence of gel cytotoxicity on mouse fibroblast cells, they performed an in vivo degradation assay conducted on mice by injecting the sterile gel into the skeletal muscle. The histological analysis of the muscles surrounding the injection site demonstrated a progressive reduction of the mild post-injection inflammation until the twentieth day, a time at which the gel showed an apparent degradation, which was complete after 30 days. In addition to this promising result in terms of a suitable degradation profile, the authors also proposed a matrix that was able to stabilize the encapsulated rabbit corneal endothelial cells. In fact, the in vivo studies on 12 rabbits’ eyes, which foresaw the instillation of a cell-incorporated solution in damaged eyes, gave appreciable results. The corneal endothelium was successfully reconstituted, and then the scaffold appeared effectively useful in corneal tissue engineering [154].

Following the aforementioned authors, Wright and coworkers developed corneal-epithelial-cells-loaded hydrogels with different degrees of alginate oxidation (1.2%, 2% and 5%), with the aim of evaluating changes in matrices’ properties as a function of oxidation [155]. The hydrogels were characterized with and without collagen IV, incorporated as a key ECM protein. They discovered that the major oxidation degree affected the viability of corneal epithelial cells and that this feature was even further enhanced in the presence of collagen IV. In detail, 2% oxidized alginate hydrogel supported cell viability in an analogous manner compared to the unmodified one and showed almost the same size in pore diameter, while the 5% oxidized hydrogel displayed internal pores with wider diameters [155]. Another interesting finding was related to stiffness of those matrices. From rheological measurements, the mean compression modulus decreased with increasing degree of oxidation. Hence, the oxidation of alginate with sodium periodate led to softer matrices than unmodified ones, thus recording another advantage of the realized scaffold [155].

In conclusion, OA represents a valid example of an advantageous alginate modification, thanks to its ability to improve not only the biodegradability of derived hydrogels but also mechanical properties and cell viability.

In Table 2, some noteworthy considerations about variable properties of alginate-based materials in corneal TERM field are summarized.

## 5. Conclusions and Future Perspectives

The lack of cornea donors has led to the growing need to satisfy the demand with engineered materials in the attempt to restore visual functions of people affected by corneal alterations. Autologous and allogeneic limbal stem cells transplants are used to date in clinical practice and allow the avoidance of the use of cadaveric grafts, but they are not free from drawbacks due to the frequent difficulties in isolating some stem cell populations, such in the case of endothelium, which is missing in a clear stem cell population.

Over recent years, the use of decellularized cornea, collagen, fibroin and other materials have been deeply investigated as efficient compounds to be exploited for tissue engineering and regeneration, and they showed interesting properties. However, considering their use as a material for corneal regeneration, it is also advisable to account for their numerous limitations in this field. Indeed, an engineered cornea requires specific properties of transparency, biocompatibility, biodegradability, adhesiveness, stability, mechanical properties and the possibility of accommodating cells, which are often not fully satisfied. To date, none of the investigated materials have been shown to have all the requirements to be considered suitable for a clinical scale-up in the field of corneal engineering and regeneration.

Alginate emerged as a valid alternative for biomedical application thanks to its versatility in the adaptation of biophysical properties, supported by its ECM-like structure and low immunogenicity. Nowadays, improvements in biomaterials’ regenerative medicine have enabled new strategies with which alginate may come much closer to being an almost ideal candidate for corneal regeneration. Controlling their mechanical and degradation features, alginate hydrogels could be fine-tuned to suit the essential requirements for the corneal TERM field. The merger with the protein originating from the ECM (gelatin or collagen) allows the enhancement of cell adherence, proliferation and viability in alginate networks. The chemical application of chelating agents in post-crosslinking phases, as well as the oxidation of the original structure of the alginate, increase its biodegradability, whose rate can be modulated according to tissue and therapeutic needs, but also as a function of the desired drug kinetic release. The conjugation of alginate with synthetic polymers, in the form of nanofiber (PCL) photo-crosslinkable material (GelMA), can refine cell orientation while also attribute to hydrogels strength and resilience characteristics but still ensuring a light refraction/transmission balance comparable to that of native cornea. On the contrary, the already optimal characteristics of the biocompatibility and transparency of alginate can be shared to improve the application of materials that do not have them by themselves.

A further emerging benefit in the use of alginate hydrogels belongs to the versatility of their manufacture from electrospinning, 3D-bioprinting and the cell-sheet-formation process easily, as well as their use in the production of conventional injectable hydrogels on a laboratory scale.

While promising results have already been obtained, the possibility to use alginate to realize a scaffold for cells on a large scale requires further confirmation. In the future, the use of alginate in corneal tissue engineering will be the subject of many successes, as well as failures, but, to date, its characteristics are promising and not negligible in the panorama of corneal TERM.

## Figures and Tables

**Table 1:** Summary of the main properties of alginate conjugates, techniques employed in their manufacture and related advantages for corneal tissue engineering.

Combined Materials	Target	Research Model				
		Manufacturing Technique	Characterization Steps	Experimental Studies	Advantages Achieved	Ref.
Alg-PLC	-	Electrospinning	- Mechanical testing - Porosity	-	- Fibrous-like structure - Enhanced robustness	[146]
	Cornea wound healing	Electrospinning	- Morphology - Thickness measurement - Optical transmission - Suture retaining test	-	- Enhanced mechanical properties - High transparency	[147]
Alg-Gel	Entire cornea	Electrospinning	- Morphology - Crosslinking studies - Mechanical testing - Optical transmission	- Ex vivo on porcine cornea	- Enhanced mechanical properties - Inexpensive and natural construct	[148]
Alg-GelMA	Corneal stroma	3D Bioprinting	- Bioinks optimization - Morphology - Transparency evaluation	- In vitro live/dead - Assay of human corneal stromal cells	- Enhanced cell viability - Transparency retained - Preservation corneal shape	[149]
Alg-RGD	Corneal epithelium	Cell sheets	- Morphology - Gel-sol optimization	- In vitro live/dead - Assay of human corneal epithelial cells - Immunostaining	- Extent cell attachment - High viability	[150]
Alg-SNF-GelMA	Corneal stroma	Micropatterned membranes	- Mechanical properties - Adhesive test - Morphology - Degradation-rate evaluation	- Ex vivo adhesion test on fresh sheepskin - In vitro MTT and live/dead tests on human stromal cells	- Good wettability - Adjustable mechanical properties - High transparency - High adhesion strength - Suitable degradation rate - Orientation of cells	[151]
OA-CMCTS	Corneal alkali burns	In situ forming hydrogel	- Optical transmission - Morphology - Swelling ability - Degradation-rate evaluation	- In vitro MTT assay on mouse fibroblast and LSCs - In vivo degradation assay on Kunming mice - In vivo efficacy on - New Zealand white rabbit eyes	- High swelling ability - High transparency - Absence of cytotoxicity - Suitable degradation rate - Marked and rapid reconstruction of injured cornea	[152]
Alg-Coll-Gel	Corneal epithelium	3D bioprinting	- Bioinks optimization - Thickness measurements - Optical transmission - Degradation-rate evaluation - Morphology	- In vitro live/dead and proliferation assay on human corneal epithelial cells	- Fast and tunable degradation - High transparency - Increased cell viability	[153]
OA-CTS	Corneal endothelium	In situ forming hydrogel	-	- In vitro MTT assay on L929 mouse fibroblast cells - Ex vivo histocompatibility assay on New Zealand white rabbits - In vivo degradation assay in Kunming mice	- Suitable degradation rate - Stabilization of cells - Successful reconstruction of endothelium	[154]
OA-Coll	Corneal epithelium		- Degree of oxidation - Porosity degree - Exclusion chromatography - Immunoblotting - Rheological measurements - Morphology	- In vitro Trypan blue exclusion assay on bovine LECs and human corneal epithelial cell	- Enhanced cell viability - Tunable mechanical properties - Predictable degradation rate	[155]

Alg = alginate; OA = oxidized alginate; PCL = polycaprolactone; Gel = gelatin; GelMA = gelatin methacrylated; RGD = Arg-Gly-Asp motif; CMCTS = carboxymethyl chitosan; Coll = collagen; LSCs = limbal stem cells; LECs = limbal epithelial cells.

**Table 2:** Variable properties of alginate-based materials to be considered and highlighted in corneal TERM applications.

Properties	Noteworthy Considerations
Strength and stiffness	Mechanical properties may be tunable as a function of the intended aim. The blend with electrospun fibers of both synthetic and natural polymers reinforce alginate-based materials, without compromising their transparency [147,148]. The use of oxidized alginate can lead to softer matrices [155]. The natural shape of the cornea has to be maintained for constructs [149].
Degradation time	The degradation rate of alginate composites can be modulated from three days to around two weeks by changing the molar ratios between alginate and chelating agents (e.g., sodium citrate) [153]. Hydrogels of oxidized alginate can be degraded in vivo after 30 days [154]. The rate of degradation seems to be directly proportional to the corneal epithelial cell viability [155].
Crosslinking methods	Ionic crosslinking (with CaCl_2_) is the most-common method, but it can slow down the degradation rate [150,153,166]. The chemical crosslinking of gel blends (with EDC-NHS) allows the finer control of the hydrogels’ physical characteristics, but it could affect cell survival [148]. Blending with photo-crosslinkable polymers ensures transparency but requires a long time for dialysis [151]. The self-crosslinking of oxidized alginate is obtainable according to the functional group of the other blending components [152]

CaCl_2_ = calcium chloride; Gel = gelatin; EDC = 1-ethyl-3-(dimethyl-aminopropyl)carbodiimide hydrochloride; NHS = N-hydroxyl succinimide.

## Data Availability

No new data were created or analyzed in this study. Data sharing is not applicable to this article.
